# Effective gene expression in the rat dorsal root ganglia with a non-viral vector delivered via spinal nerve injection

**DOI:** 10.1038/srep35612

**Published:** 2016-10-17

**Authors:** Ming-Fong Chang, Jung-Hsien Hsieh, Hao Chiang, Hung-Wei Kan, Cho-Min Huang, Luke Chellis, Bo-Shiou Lin, Shi-Chuen Miaw, Chun-Liang Pan, Chi-Chao Chao, Sung-Tsang Hsieh

**Affiliations:** 1 Department of Anatomy and Cell Biology, College of Medicine, National Taiwan University, Taipei, 10051, Taiwan; 2 Departments of Surgery, National Taiwan University Hospital, Taipei, Taiwan; 3 Department of Brain and Cognitive Sciences, Massachusetts Institute of Technology, USA; 4 Department of Graduate Institute of Immunology, College of Medicine, National Taiwan University, Taipei, 10051, Taiwan; 5 Department of Graduate Institute of Molecular Medicine, College of Medicine, National Taiwan University, No. 7 Chung-Shan South Road, Taipei, 10002, Taiwan; 6 Departments of Neurology, National Taiwan University Hospital, Taipei, Taiwan; 7 Department of Graduate Institute of Brain and Mind Science, College of Medicine, National Taiwan University, Taipei, 10051, Taiwan; 8 Departments of Clinical Center for Neuroscience and Behavior, National Taiwan University Hospital, Taipei, Taiwan; 9 Department of Graduate Institute of Clinical Medicine, College of Medicine, National Taiwan University, Taipei, 10051, Taiwan

## Abstract

Delivering gene constructs into the dorsal root ganglia (DRG) is a powerful but challenging therapeutic strategy for sensory disorders affecting the DRG and their peripheral processes. The current delivery methods of direct intra-DRG injection and intrathecal injection have several disadvantages, including potential injury to DRG neurons and low transfection efficiency, respectively. This study aimed to develop a spinal nerve injection strategy to deliver polyethylenimine mixed with plasmid (PEI/DNA polyplexes) containing green fluorescent protein (GFP). Using this spinal nerve injection approach, PEI/DNA polyplexes were delivered to DRG neurons without nerve injury. Within one week of the delivery, GFP expression was detected in 82.8% ± 1.70% of DRG neurons, comparable to the levels obtained by intra-DRG injection (81.3% ± 5.1%, p = 0.82) but much higher than those obtained by intrathecal injection. The degree of GFP expression by neurofilament(+) and peripherin(+) DRG neurons was similar. The safety of this approach was documented by the absence of injury marker expression, including activation transcription factor 3 and ionized calcium binding adaptor molecule 1 for neurons and glia, respectively, as well as the absence of behavioral changes. These results demonstrated the efficacy and safety of delivering PEI/DNA polyplexes to DRG neurons via spinal nerve injection.

Sensory neuropathy, or the degeneration of dorsal root ganglia (DRG) sensory neurons and their nerves, occurs in various diseases ranging from diabetes to the toxicity of chemotherapeutic medications^1^,^2^. Patients typically suffer from symptoms of reduced sensation and neuropathic pain. However, the molecular mechanisms of neuropathic pain remain unclear. The delivery of gene constructs into DRG neurons is a major approach for investigating the molecular mechanisms of injury-induced neuropathic pain and designing specific therapies for nerve regeneration. Non-viral gene transfection provides an alternative approach for delivering gene constructs to DRG neurons. Several methods of nanocarriers have been developed for non-viral gene transfection, such as cationic lipids and polyethylenimine (PEI)^3^,^4^. However, the transfection efficiencies of neurons using these methods are low^3^,^5^,^6^,^7^,^8^,^9^. Thus, the development of new delivery methods for improved transfection efficiency in DRG neurons is crucial for gene therapy studies of sensory neuropathies.

There are several routes to deliver molecules for manipulating gene expression in DRG neurons, such as intraplantar^10^,^11^,^12^, intrathecal and intra-DRG injection^13^,^14^,^15^,^16^. Cutaneous injection is an easy approach, but multiple injections are required because a spinal nerve is distributed over a large area. Efficiency constitutes a major concern for intrathecal injection, in that a large proportion of injected molecules are retained at the pia of the spinal cord^4^. Specific neuronal targeting by intrathecal injection is also a critical issue, as the expression of an injected non-viral construct, for example, may extend to astrocytes and microglia of the spinal cord in addition to neurons^4^. Ectopic expression of DRG proteins in the motor neurons of the spinal cord may potentially produce unwanted effects^17^. Considering that these treatments may potentially affect motor nerve function, direct injection into the DRG presents a straightforward and appealing approach; however, it requires the extensive removal of bony structures that could potentially damage DRG neurons and their nerve fibers^13^,^18^,^19^. Thus, alternative approaches of gene delivery to DRG neurons will facilitate gene therapy studies in sensory neuropathies. These issues prompted us to develop an approach that can deliver gene constructs specifically to the DRG without causing an immune response or causing damage to the DRG and its nerve fibers.

The risk of variable expression levels of transduced genes in the DRG is another issue^13^,^16^, given that the DRG contains neurons of various sizes and phenotypes^20^,^21^,^22^, such as proprioceptors or thermal nociceptors^23^,^24^,^25^,^26^. Neurofilaments and peripherin are commonly used to assess large and small neurons, respectively^21^,^22^,^27^,^28^,^29^,^30^,^31^. The sizes of DRG neurons form a continuous spectrum; it is not yet known what percentage of DRG neurons express both neurofilaments and peripherin. By combining the readily appreciated differences between neuronal soma and nerve fibers under differential interference contrast optics with the application of molecular markers specific for subsets of DRG neurons, we wish to determine the targeting specificity of our approach down to the single-neuron levels.

To address these issues, we aimed to develop a new approach for delivering gene constructs via spinal nerve injection to DRG neurons. This method was further compared with intra-DRG injection and intrathecal injection in the context of efficiency and safety, including transfection efficiency and the immune reaction induced in the DRG.

## Results

### Evaluation of delivering gene constructs into DRG: dye injection

**Figure 1:**
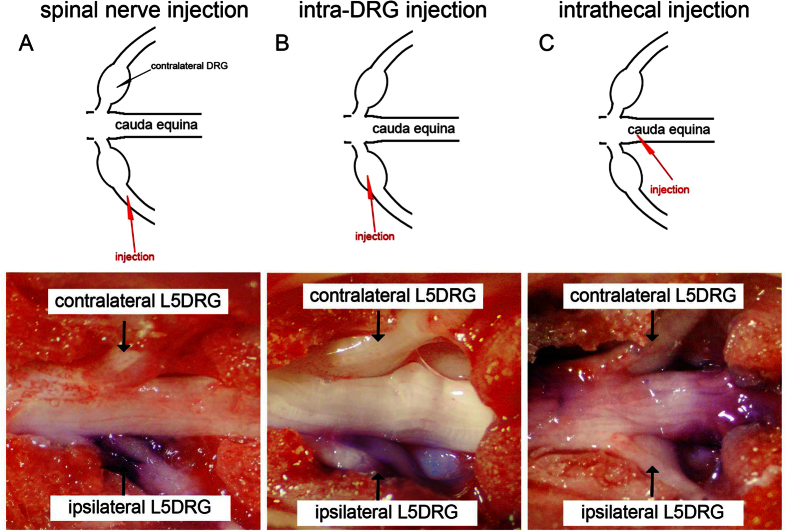
Introducing dye into dorsal root ganglia (DRG) neurons via different methods. The feasibility of different injection routes was assessed with a toluidine blue dye injection test using spinal nerve injection, intra-DRG injection, and intrathecal injection. The diagrams for each corresponding photo are in the upper panel. The diagram illustrates the injection site (red arrowhead) for spinal nerve injection (**A**), intra-DRG injection (**B**), and intrathecal injection (**C**). (**A**) In the spinal nerve injection approach, 3 μl of toluidine blue dye was injected in the L5 spinal root and ipsilateral L5 DRG. There was no dye in the contralateral L5 DRG. (**B**) The injected dye was apparent in the ipsilateral L5 DRG after intra-DRG injection. (**C**) There was only limited dye observed in the L5 DRG after the intrathecal injection of 10 μl toluidine blue. The majority of toluidine blue retained in the cauda equina.

To deliver gene constructs into the DRG, we developed a spinal nerve injection approach. We used 3 examinations to assess the feasibility, efficiency, and safety of this approach. This method was first validated with dye injection through the L5 spinal nerve, and it was compared with intrathecal injection and intra-DRG injection methods (Fig. 1A–C), which were used as the positive controls. After the injection of 3 μl toluidine blue into the L5 spinal nerve, the dye appeared in the L5 DRG and the L5 spinal nerve on the ipsilateral side, without extension into the contralateral DRG or other spinal nerves (Fig. 1A) compared to the intrathecal (Fig. 1B) and intra-DRG injection groups (Fig. 1C).

### Demonstration of green fluorescent protein (GFP) expression in the dorsal root ganglia (DRG) after spinal nerve injection

**Figure 2:**
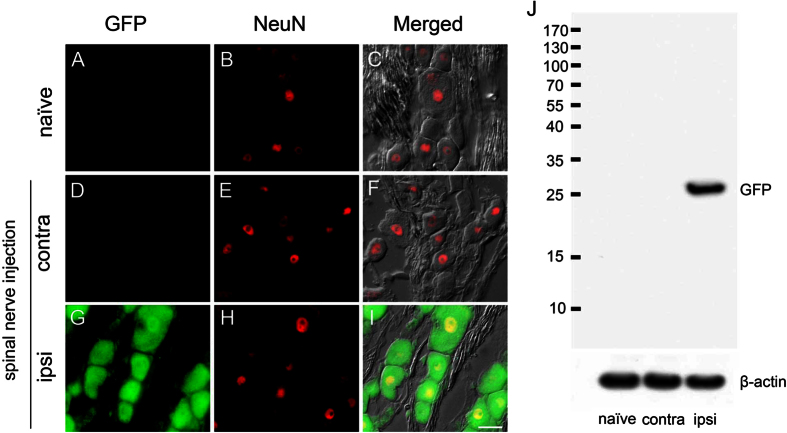
Expression of green fluorescent protein (GFP) in the dorsal root ganglia (DRG) via spinal nerve injection. The expression of GFP in DRG neurons was assessed with double immunofluorescence staining with anti-GFP and anti-neuron nuclear binding protein (NeuN) as well as differential interference contrast (DIC) microscopy at one week post-operation. (**A**–**F**) No GFP(+) neurons were detected in the naïve group or the contralateral DRG. (**G**–**I**) There were GFP(+) neurons in the ipsilateral L5 DRG after spinal nerve injection. GFP immunoreactivity appeared only in the neuronal nucleus and cytoplasm. (**J**) GFP expression in the ipsilateral L5 DRG was confirmed by immunoblotting after spinal nerve injection. Correct molecular weight ≅ 27 kDa. The full-length blots were presented in supplementary information (Fig. S3). Bar, 25 μm.

To exclude the possibility that GFP-like signals in the DRG neuron are actually caused by autofluorescence, we performed double-labeling immunofluorescence studies in the DRG with antibodies against GFP and NeuN, as a neuronal marker, one week after delivering polyethylenimine mixed with DNA plasmids (PEI/DNA polyplexes) that contained the gene encoding green fluorescent protein (Fig. 2A–F). There was no GFP expression in the naïve group or on the contralateral side of the injection group. On the ipsilateral side of the injection group, GFP appeared in the neuronal soma and nucleus (Fig. 2G–I). To examine GFP expression biochemically, we performed western blotting on the DRG. Western blots demonstrated that the GFP expression was specific on the ipsilateral DRG of the spinal nerve injection group compared with the naïve DRG and contralateral side DRG (Fig. 2J). The results excluded the possibility of GFP expression due to autofluorescence and showed the specific pattern of GFP expression immunohistochemically and biochemically.

### Assessment of glial cell activation in the dorsal root ganglia (DRG) after intrathecal, spinal nerve and intra-DRG injection

**Figure 3:**
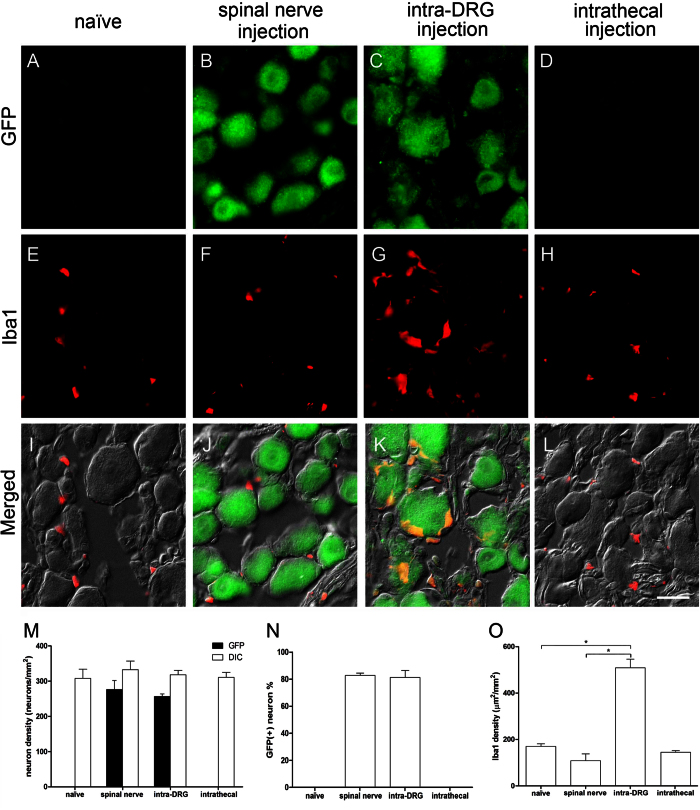
Expression of green fluorescent protein (GFP) and ionized calcium binding adaptor molecule 1 (Iba1) in the dorsal root ganglia (DRG) following different injection approaches. DRG neurons were stained with anti-GFP (1st row) and Iba1 (2nd row), and the fluorescence staining and differential interference contrast (DIC) images were merged (3rd row) after injection with the constructs. The naïve group (1st column), the spinal nerve injection group (2nd column), the intra-DRG injection group (3rd column), and the intrathecal injection (4th column) group were compared. (**M**–**N**) The graph compares the transfection efficiency of different approaches based on the total GFP(+) neuronal density (**M**) and ratio (**N**). (**O**) The graph compares the Iba1(+) area density in all groups. Bar, 25 μm *Statistically significant at *p* < 0.05.

To determine the transgene expression and glia activation of different routes in the DRG, one week after delivering the PEI/DNA polyplexes, we performed immunostaining for GFP and Iba1, as a glial activation marker, on DRG sections. The results demonstrated increased GFP expression in the DRG neurons in the spinal nerve and intra-DRG injection groups (Fig. 3B,C) compared to the naïve and intrathecal injection groups (Fig. 3A,D). The transfection efficiency was similar between the spinal nerve injection group and the intra-DRG injection group (82.8% ± 1.7% vs. 81.3% ± 5.1%, *p* = 0.82, Fig. 3M,N) and was much higher than that of the intrathecal injection and naïve groups (0% ± 0%, *p* < 0.01). In addition, there were no Iba1(+) cells in the naïve, intrathecal, and spinal nerve injection groups (Fig. 3E,F,H), but these cells were present in the intra-DRG injection group (Fig. 3G). Quantitatively, the Iba1 expression of the intra-DRG group was higher than that of the spinal nerve injection group (509.2 ± 37.12 μm^2^/mm^2^ vs. 108.3 ± 29.71 μm^2^/mm^2^, *p* < 0.001, Fig. 3O) and the naïve group (509.2 ± 37.12 μm^2^/mm^2^ vs. 170.4 ± 21.67 μm^2^/mm^2^, *p* = 0.002, Fig. 3O).

### Quantitation of gene expression patterns in DRG neurons

**Figure 4:**
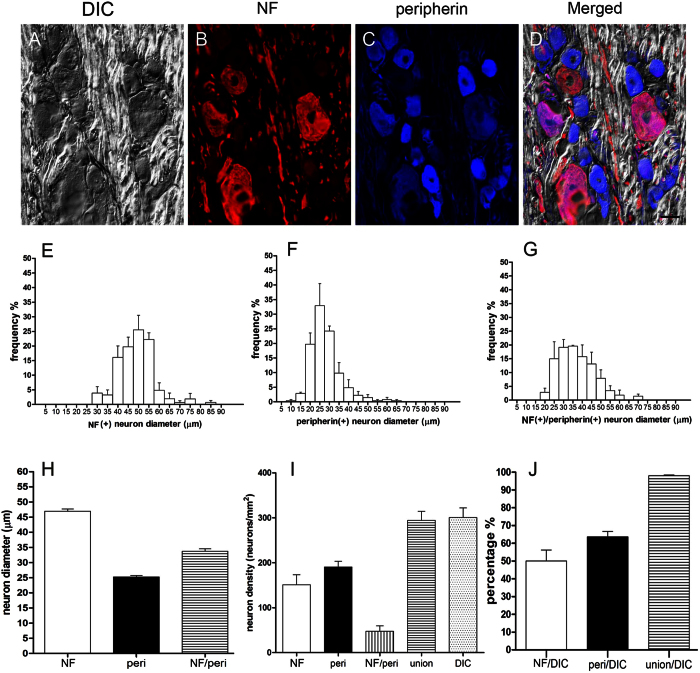
Quantitation of dorsal root ganglia neurons. (**A**–**D**) Dorsal root ganglia (DRG) neurons were immunostained for neurofilaments (NF) and peripherin (peri) and observed under differential interference contrast (DIC) microscopy. (**E**–**J**) DRG neurons were quantified according to immunostaining, neuron diameter, and neuron density. **union** = neurofilament(+) **+** peripherin(+) **−**neurofilament (+)/peripherin(+). Bar, 25 μm.

To further examine the targeting specificity of DRG neuronal subtypes by our spinal nerve injection-based gene delivery, we developed a comprehensive method for quantifying total DRG neurons by combining images of differential interference contrast (DIC) microscopy (Fig. 4A) and immunostaining with anti-neurofilament antisera for large-diameter neurons (Fig. 4B) and anti-peripherin for small-diameter neurons (Fig. 4C). The diameters of neurofilament(+) neurons (Fig. 4B,E) were larger than those of peripherin(+) neurons (Fig. 4C–F). The diameters of neurofilament(+) neurons, peripherin(+) neurons and neurofilament(+)/peripherin(+) neurons were 47.0 ± 0.7 μm, 25.3 ± 0.5 μm and 33.8 ± 0.8 μm, respectively, forming 3 subtypes of DRG neurons (Fig. 4E–H). We then determined whether double-immunolabeling of peripherin and neurofilaments identified all DRG neurons with the following formula:


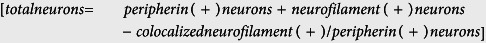


Using this method, we found that the DRG neuronal number revealed by double-immunostaining was comparable to that counted by DIC microscopy (294.3 ± 19.9 vs. 300.6 ± 21.4 neurons/mm^2^, *p* = 0.838, Fig. 4I–J).

### Expression patterns of GFP in DRG neurons after spinal nerve injection and intra-DRG injection

**Figure 5:**
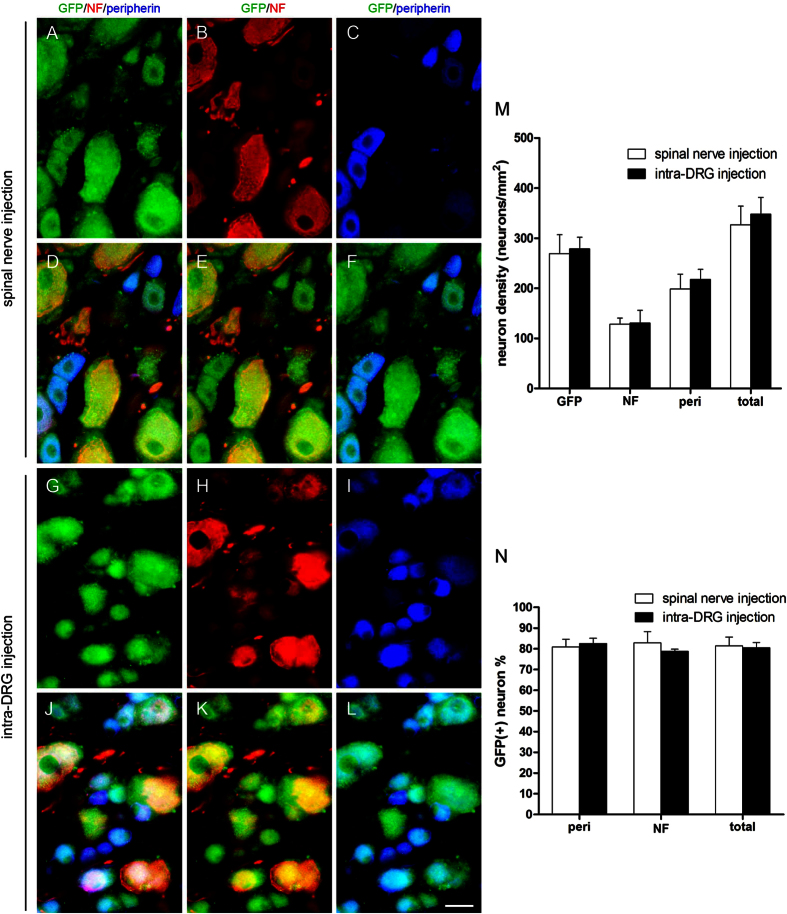
Expression of green fluorescent protein (GFP) in the dorsal root ganglia (DRG) after gene constructs were delivered via spinal nerve injection and intra-DRG injection. Immunofluorescent staining of DRG sections was performed with anti-GFP (GFP, green), anti-neurofilament (NF, red) and anti-peripherin (peri, blue) antibodies in the spinal nerve injection group (**A**–**F**) and the intra-DRG injection group (**G**–**L**). The results were quantified, and the neuronal density (**M**) and ratio (**N**) were analyzed. Bar, 25 μm.

We then applied this quantitation strategy to investigate the patterns of GFP expression in the DRG neurons of different subtypes by conducting triple-labeling immunofluorescence staining (GFP, peripherin, neurofilament) one week after delivery (Fig. 5A–L). These results demonstrated the efficiency of GFP expression in both neurofilament(+) and peripherin(+) neurons. The transfection efficiency of GFP expression was similar in both neurofilament(+) neurons and peripherin(+) neurons in the spinal nerve injection group (82.8% ± 5.5% vs. 80.9% ± 3.7%*, p* = 0.66, Fig. 5M,N), and these levels were similar to the transfection efficiencies of neurofilament(+) neurons and peripherin(+) neurons in the intra-DRG injection group (78.8% ± 1.03% vs. 82.5% ± 2.6%, *p* = 0.25, Fig. 5M,N). These results indicated that the gene expression driven by spinal nerve injection was efficient and universal to different types of DRG neurons.

### Safety assessment of gene delivery though spinal nerve injection: multi-modality evaluation

The above observations demonstrated the feasibility and efficiency of delivering gene constructs through spinal nerve injection, which was comparable to those observed with intra-DRG injection and superior to the intrathecal injection methods. We next conducted safety assessments of our spinal nerve injection approach and intra-DRG injection, including: (1) behavioral and physiological tests, (2) the expression of neuronal injury markers, (3) patterns of microglia and astrocyte activation, and (4) pathologic studies of nerves and their terminals. In all these comparative examinations, axotomy by transecting the sciatic nerve and crush by crushing spinal nerve were used as a positive control for nerve injury.

**Figure 6:**
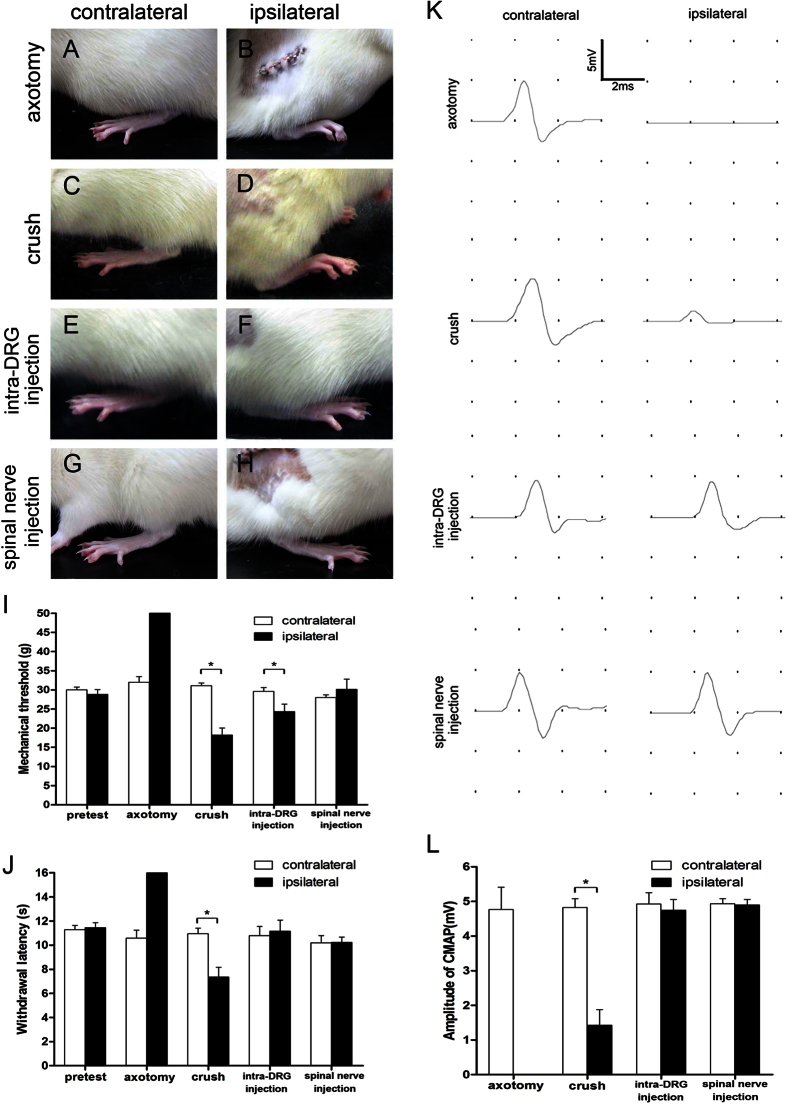
Change in hindlimb posture, behavioral tests, and nerve conduction studies after spinal nerve injection. The effects of spinal nerve injection (spinal nerve injection group) compared to sciatic nerve transection (axotomy group), spinal nerve crush (crush group) and intra-DRG injection (intra-DRG injection group) one week after operation were evaluated, including hindlimb posture (**A**–**H**), behavioral tests of hindpaw mechanical threshold (**I**), and hindpaw thermal withdraw latency (**J**), as well as nerve conduction studies of the compound muscle action potential (CMAP) on stimulating the sciatic nerve and according the plantar muscles amplitude (**K,L**). (**A–D**) The ipsilateral hindpaw of rats exhibited typical posture with flexion of toes one week post-operation in the axotomy group and crush group. (**C–H**) There was no postural change in the intra-DRG and the spinal nerve injection groups. The hindpaw was placed flat; the toes on the ipsilateral side spread similarly to those on the contralateral side. (**I**) The mechanical thresholds were elevated on the ipsilateral hindpaw of the axotomy group but decreased on the ipsilateral hindpaw of the crush group and intra-DRG injection groups. There was no alteration of mechanical threshold in the spinal nerve injection group. (**J**) The withdrawal latencies were increased on the ipsilateral hindpaw of the axotomy group, decreased on the ipsilateral hindpaw of the crush group, and there was no thermal hyperalgesia in the intra-DRG and spinal nerve injection groups. (**K**) CMAP could not be evoked on the ipsilateral side of the axotomy group and CMAP amplitude was decreased on the ipsilateral side of the crush group. Similar CMAP amplitude could be evoked in both the contralateral and ipsilateral plantar muscles in the intra-DRG and the spinal nerve injection groups. (**L**) CMAP amplitude was reduced on the ipsilateral side of the axotomy and the crush groups, but there was no difference in the CMAP amplitudes on both sides in the intra-DRG and the spinal nerve injection groups. *Statistically significant at *p* < 0.05.

Peripheral nerve injury induced mild elevation of the hindpaw in neuropathic pain studies^32^,^33^. We evaluated postural changes of the hindpaw one week after the procedure (Fig. 6A–H). In the spinal nerve injection group and intra-DRG injection, there was no change in the posture of the hindpaw compared to that of the axotomy group and the crush group, which were mildly elevated with toes flexed.

Mechanical thresholds and thermal withdrawal latencies were on the hindpaws measured to assay changes in sensory nerve function. Mechanical thresholds and thermal withdrawal latencies of contralateral side in all groups were similar to those of the pretest group without significance difference (Fig. 6I,J). In the axotomy group, the mechanical thresholds were markedly increased (50.0 ± 0.0 vs. 31.98 ± 1.45 g, *p* < 0.001, Fig. 6I), and the thermal latencies were prolonged (16.0 ± 0.0 vs. 10.58 ± 0.66 s, *p* < 0.001, Fig. 6J) on the ipsilateral side compared to the contralateral side at one week post-operation. There was mechanical allodynia in the crush and intra-DRG injection groups with reduced mechanical thresholds on the ipsilateral side compared to the contralateral side at one week post-operation (18.2 ± 1.84 vs. 31.08 ± 0.73 g, *p* < 0.001 and 24.28 ± 1.99 vs.29.6 ± 0.99 g, *p* = 0.013, Fig. 6I). There was thermal hyperalgesia in the crush group with decreased thermal latencies on the ipsilateral side compared to the contralateral side at one week post-operation (7.35 ± 0.81 vs. 10.95 ± 0.46 s, *p* = 0.032, Fig. 6J). There was no difference between the injected side and the contralateral side of the spinal nerve injection group in mechanical thresholds (30.15 ± 2.64 vs. 27.98 ± 0.76 g, *p* = 0.45, Fig. 6I) and thermal latencies (10.23 ± 0.44 vs. 10.20 ± 0.60 s, *p* = 0.97, Fig. 6J) compared to the axotomy, crush, and intra-DRG injection groups.

Nerve conduction studies were performed to assess the motor nerve physiology. In the axotomy group, compound muscle action potential (CMAP) was absent on the operated side, in contrast to the contralateral side one week after the surgery (0.0 ± 0.0 vs. 4.76 ± 0.64 mV, *p* < 0.001, Fig. 6K,L). In the crush group, CMAP amplitude of the plantar muscles was decreased on the operated side compared to the contralateral side one week after the surgery (1.42 ± 0.44 vs. 4.8 ± 0.24 mV, p < 0.001, Fig. 6K,L). There was no difference in CMAP amplitude between the operated side and the contralateral side in the intra-DRG injection and spinal nerve injection groups (4.75 ± 0.3 vs. 4.92 ± 0.33 mV, p = 0.74 and 4.90 ± 0.15 vs. 4.93 ± 0.14 mV, *p* = 0.88, Fig. 6K,L). Taken together, we concluded that sensory and motor nerve functions were intact after spinal nerve injection.

After all operations, we carefully monitored all animals including (1) infection signs (redness and welling of tissues), and (2) tissue damage (infiltration of macrophages and expression of neuronal injury markers). In all these animals, there were no infection signs, i.e. redness and swelling of tissues and animals could freely ambulate. To further assess tissue injury, DRG sections were stained with Iba1 (a marker of macrophage infiltration) and phosphorylated neurofilaments (p-NF, as a marker of nerve injury). p-NF was expressed in nerve fibers of control animals but was detectable in neuronal cell bodies after nerve injury. Nerve injury induced expression of p-NF in the neuronal soma on the ipsilateral side DRG of the axotomy, crush, and intra-DRG injection groups (Fig. S1D,F,G,I,J,L) compared to the contralateral side and the ipsilateral side of the spinal nerve injection group (Fig. S1A,C,M,O). The Iba1 expression was increased on the ipsilateral DRG of the axotomy, crush, and intra-DRG injection groups (Fig. S1E,F,H,I,K,L) in comparison to the contralateral side and the ipsilateral side of the spinal nerve injection group (Fig. S1B,C,N,O). In addition, nerve injury induced eccentric nuclei on the ipsilateral side DRG of the axotomy, crush, and intra-DRG injection groups (Fig. S2D,F,G,I,J,L) compared to the contralateral side and the ipsilateral side of the spinal nerve injection group (Fig. S2A,C,M,O).

### Expression of activating transcription factor 3 (ATF3) after spinal nerve injection

To further explore the possible DRG injury response after spinal nerve injection, we performed immunostaining for activating transcription factor 3 (ATF3), an established nerve injury marker^34^,^35^. ATF3 was strongly expressed in the DRG and ventral horn motor neurons of the axotomy and crush group (Fig,J,M,N,Q,R,U,V). In the intra-DRG injection group, ATF3 was expressed in DRG neurons (Fig,S). In contrast, in the spinal nerve injection group, no ATF3 immunoreactivity could be detected (Fig,P,T,X).

**Figure 7:**
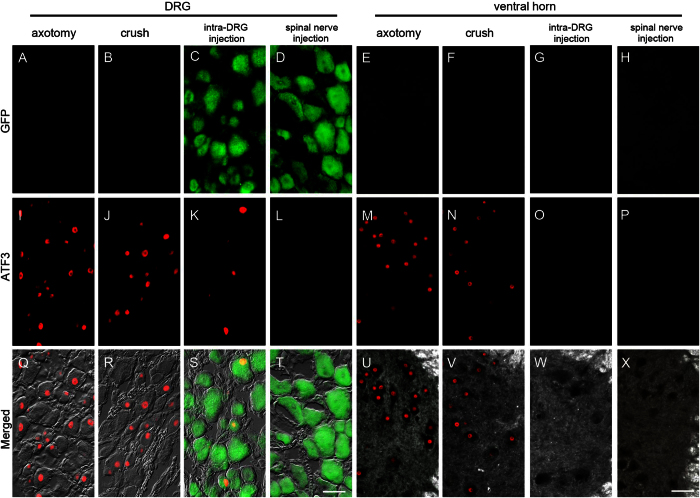
Expression of activating transcription factor 3 (ATF3) and green fluorescent protein (GFP) in the dorsal root ganglia (DRG) and spinal cord after spinal nerve injection (injection group) in comparison with sciatic nerve transection (axotomy group), spinal nerve crush (crush group), and intra-DRG injection (intra-DRG injection group). Double-labeling immunofluorescence staining was performed for GFP (**A**–**H**) and ATF3 (**I**–**P**) in the DRG (1st, 2nd, 3rd and 4th columns) and spinal cord (5th, 6th, 7th, and 8th columns) in the axotomy (1st and 5th columns), crush (2nd and 6th columns), intra-DRG injection (3rd and 7th columns) and spinal nerve injection (4th and 8th columns) groups. Fluorescence staining and dark field images were merged (**U**–**X**). GFP was strongly expressed in the ipsilateral DRG (**C,D,S,T**) but was absent in the ventral horn (**G,H,W,X**) of the injection group. There were no GFP(+) neurons in the axotomy and the crush group (**A,B,E,F,Q,R,U,V**). ATF3 expression was evident in the ipsilateral DRG and ventral horn of the axotomy, crush and intra-DRG injection groups (**I–K,M,N,Q–S,U,V**), in contrast to the spinal nerve injection group (**L,T,P,X**). Bar, 50 μm.

There were no GFP(+) motor neurons in the ventral horns of the intra-DRG injection and spinal nerve injection group (Fig. 7G,H,W,X), suggesting that the GFP(+) gene constructs were specifically delivered to the DRG without ectopically targeting the motor neurons of the ventral horn.

### Assessment of glial cells and macrophage activation in the spinal nerve after spinal nerve injection

**Figure 8:**
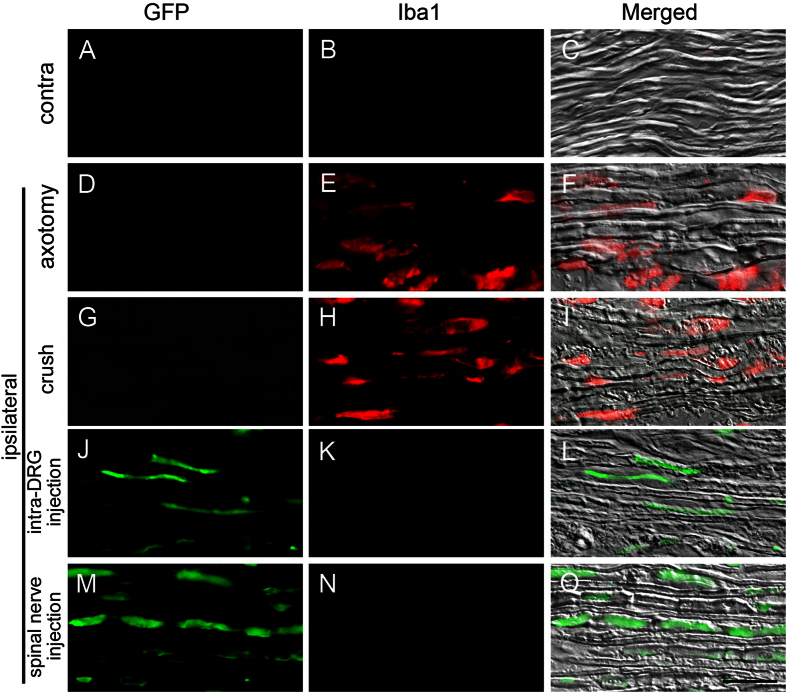
Expression of ionized calcium binding adaptor molecule 1 (Iba1) and green fluorescent protein (GFP) in the spinal nerve after spinal nerve injection (injection group) compared to sciatic nerve transection (axotomy group), spinal nerve crush (crush group) and intra-DRG injection (intra-DRG injection group). Double-labeling immunofluorescence staining was performed for GFP (1st column) and Iba1 (2nd column). Fluorescence staining and differential interference contrast (DIC) images were merged (3rd column) to visualize the spinal nerves of the contralateral side (**A**–**C**) and of the ipsilateral side in the axotomy (**D**–**F**), crush (**G**–**I**), intra-DRG injection (**J**–**L**) and spinal nerve injection (**M**–**O**) groups. GFP immunoreactivity was present in the spinal nerves of the spinal nerve injection group but was absent in the axotomy group. Iba1 immunoreactivity was present in the spinal nerves of the axotomy group but was absent in the spinal injection group. Bar, 25 μm.

To assay whether spinal nerve injection triggered the activation of the macrophages in the spinal nerve, we examined the expression of Iba1 one week post-operation. The infiltration of Iba1(+) macrophages was increased in the spinal nerves of the axotomy and crush groups (Fig. 8E,F,H,I). In contrast, there were only minimal Iba1(+) cells in the contralateral groups (Fig. 8B,C), intra-DRG injection group (Fig. 8K,L), and the spinal nerve injection group (Fig. 8N,O).

### Assessment of astrocyte and microglia activation in the spinal cord after spinal nerve injection

**Figure 9:**
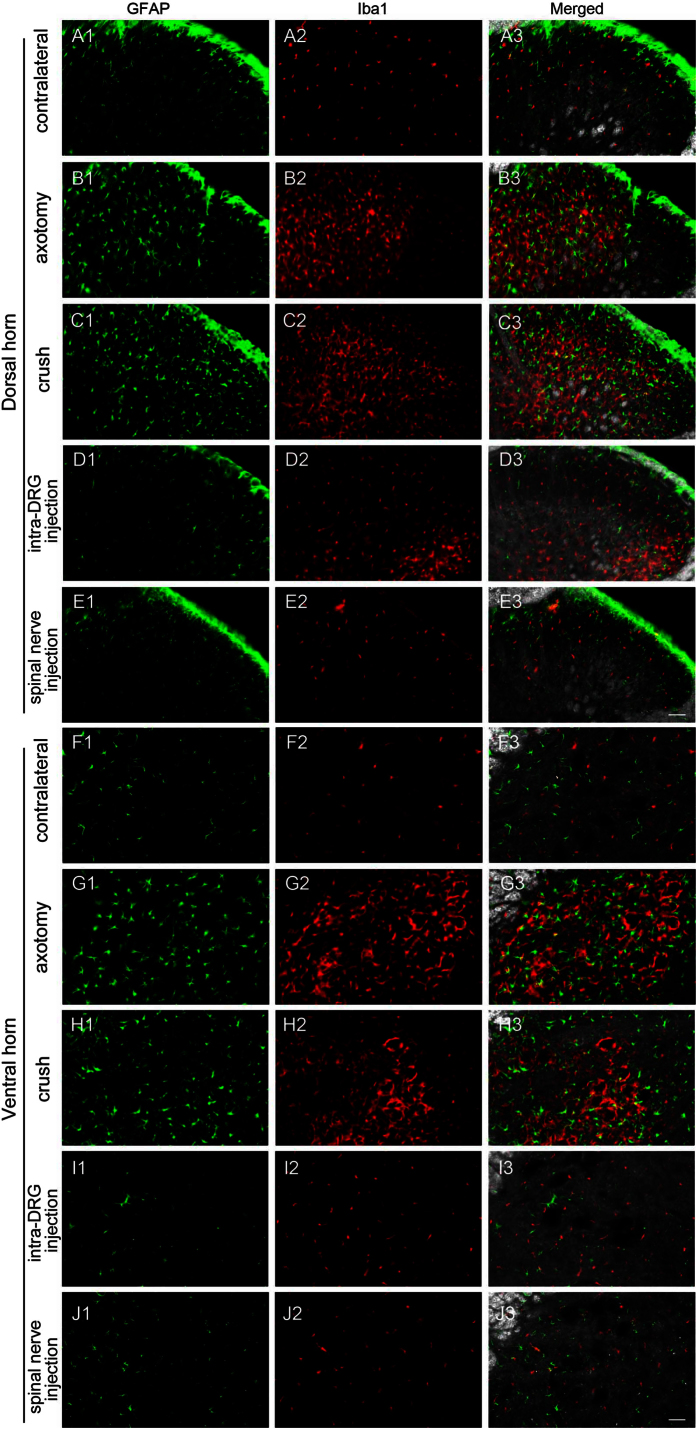
Expression of glial fibrillary acidic protein (GFAP) and ionized calcium binding adaptor molecule 1 (Iba1) in the spinal cord after spinal nerve injection (spinal nerve injection group) in comparison with sciatic nerve transection (axotomy group), spinal nerve crush (crush group), and intra-DRG injection (intra-DRG injection). Double-labeling immunofluorescence staining was performed for GFAP (1st column) and Iba1 (2nd column). Fluorescence staining and dark field images were merged (3rd column) to visualize the dorsal horn (**A**–**E**) and ventral horn (**F**–**J**). On the contralateral side of the axotomy (1st and 6th rows) and spinal nerve injection groups (5th and 10th rows), there was scanty GFAP and Iba1 immunoreactivity in the dorsal and ventral horn. Glial and microglial activation, as documented by an increase in GFAP(+) and Iba1(+) cells, was evident in the dorsal horn and ventral horn on the ipsilateral side of the axotomy and the crush group (2nd, 3rd, 6th and 7th rows). Microglial activation, as documented by an increase in Iba1(+) cells, was evident in the dorsal horn on the ipsilateral side of the intra-DRG injection group (4th row). Bar, 50 μm.

To determine whether spinal nerve injection triggered the activation of astrocytes and microglia in the spinal cord, we examined the expression of GFAP (astrocytes) and Iba1 (microglia) in the spinal cord one week post-operation. In the spinal cord (both the dorsal horn and the ventral horn), GFAP(+) astrocytes and Iba1(+) microglia were increased on the ipsilateral side of the axotomy, crush, and intra-DRG injection groups (Fig. 9B1–B3,C1–C3,D2–D3,G1–G3,H1–H3) compared to the contralateral side (Fig. 9A1–A3,F1–F3) and the spinal nerve injection group (Fig. 9E1–E3,J1–J3). These results indicated that the activation of astrocytes and microglia was minimal in the spinal cord after spinal nerve injection.

### Integrity of DRG neurons and their processes after spinal nerve injection

**Figure 10:**
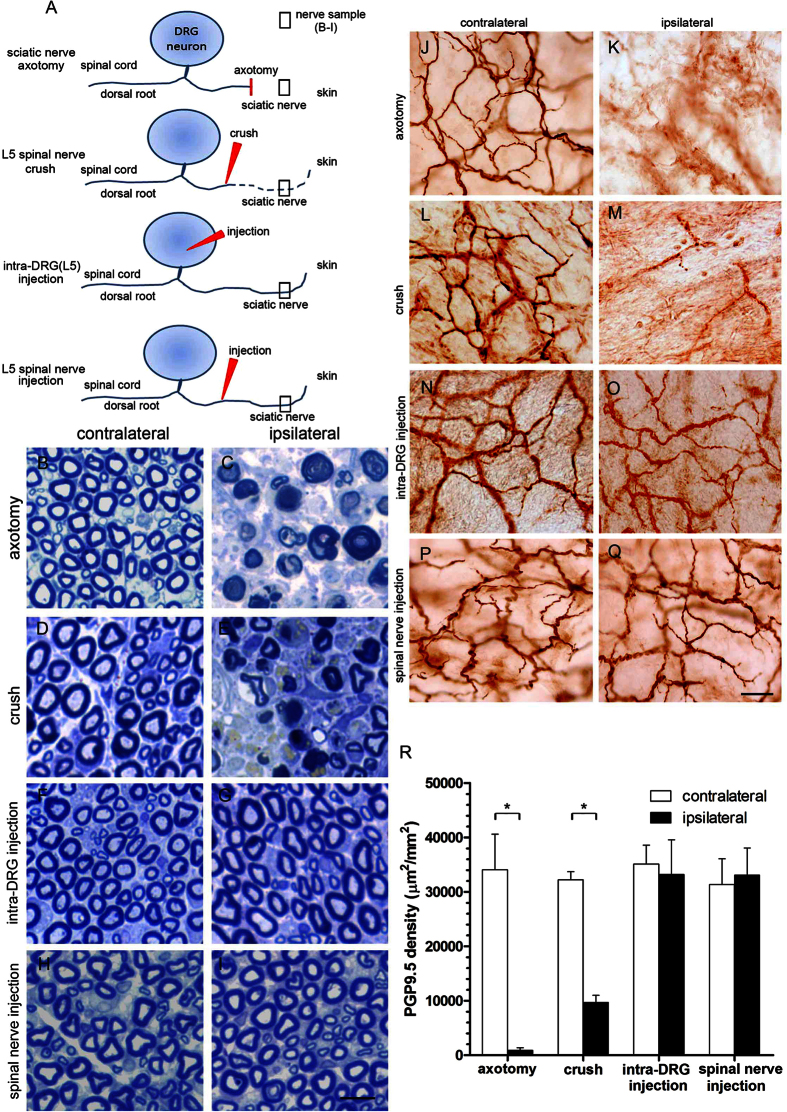
Pathology of the sciatic nerve and nerve terminals in the skin after spinal nerve injection. (**A**) The diagram illustrates the peripheral processes and terminals of dorsal root ganglia (DRG) neurons. The peripheral processes typically degenerate after sciatic nerve transection (axotomy group) and spinal nerve crush (crush group) but remained intact after intra-DRG injection (intra-DRG injection group) and spinal nerve injection (spinal nerve injection group). (**B**–**I**) Sciatic nerves at the gluteal level were processed for nerve pathologic studies after axotomy, spinal nerve crush, intra-DRG injection, and spinal nerve injection. Axonal degeneration was prominent in the axotomy group and the crush group but not in the intra-DRG injection group and the spinal nerve injection group. (**J**–**Q**) The nerve terminals of the DRG neurons in the dermis of the skin from both groups were immunostained with anti-protein gene product 9.5 (PGP9.5), a pan-neuronal marker. In the skin on the contralateral hindpaw of the axotomy group and the crush group, PGP9.5(+) nerve fibers exhibited intense linear profiles forming interlacing networks; the dermal nerve fibers degenerated on the ipsilateral skin (**J**–**M**). In the intra-DRG injection group and the spinal nerve injection group, PGP9.5(+) dermal nerve fibers appeared with similar abundance on the ipsilateral and contralateral sides (**N**–**Q**). (**R**) The graph compares the dermal nerve fiber in the axotomy, crush, intra-DRG injection and spinal nerve injection groups. Bar, 10 μm (**B**–**I**), 25 μm (**J**–**Q**). *Statistically significant at *p* < 0.05.

To explore whether the processes of DRG neurons were intact after spinal nerve injection, we examined the pathology of the sciatic nerves at the gluteal level and their terminals in the skin one week after surgery. The nerve fibers remained intact in the sciatic nerve on the ipsilateral side of the spinal nerve injection group, in contrast to the significant axonal degeneration observed in the axotomy and crush groups (Fig. 10A–I). The effects of these surgical procedures on the peripheral nerve terminals of the DRG in the epidermis were evaluated with protein gene product 9.5 (PGP9.5) immunohistochemistry. PGP9.5 is a neuronal marker that stains all nerve fibers of different phenotypes^36^. PGP9.5(+) dermal nerves presented linear profiles, forming interlacing networks in the skin of the control side (Fig. 10J,L,N,P). No PGP9.5(+) nerve fibers and decreased nerve fibers were observed in the ipsilateral side of the axotomy group and crush groups (Fig. 10J–M). In contrast, there was no difference in the dermal nerves of the injected and control sides in the intra-DRG injection group and the spinal nerve injection group (Fig. 10N–Q). This observation was verified by quantitation of dermal PGP9.5(+) nerve fibers on the ipsilateral side relative to the control side of the axotomy group (913.8 ± 463.0 vs. 34065.7 ± 6523.5 μm^2^/mm^2^, *p* = 0.003, Fig. 10R) and the crush group (9684.2 ± 1293.7 vs. 32210.9 ± 1490.7 μm^2^/mm^2^, p < 0.001, Fig. 10R). In the intra-DRG injection and spinal nerve injection groups, there were no difference between the ipsilateral and contralateral sides (33192.1 ± 6353 vs. 35097.8 ± 3484 μm^2^/mm^2^, *p* = 0.40 and 33047.0 ± 5016.9 vs. 31320.6 ± 4731.4 μm^2^/mm^2^, *p* = 0.41, Fig. 10R).

## Discussion

This report established the efficacy, specificity and safety of spinal nerve injection as a novel approach to deliver PEI/DNA polyplexes into the DRG. Gene expression by this method is restricted to DRG sensory neurons, as demonstrated by a lack of GFP expression in the motor neurons of the ventral horn or glial cells in the DRG and the spinal cord. This approach achieved high transfection efficiency (82.8% ± 1.70%), comparable to direct intra-DRG injection. In addition, our results demonstrated that delivering the gene constructs into the DRG via spinal nerve injection did not induce glia activation compared to the intra-DRG injection group. The analysis of neuronal patterns indicated that this spinal nerve injection approach could deliver gene constructs to all subtypes of DRG neurons with similar efficiency. Furthermore, the safety of this approach was confirmed by (1) the absence of behavioral abnormalities, (2) the absence of ATF3 expression in DRG neurons and the ventral horns, (3) the absence of glia and/or macrophage activation in the DRG, spinal nerves, and spinal cord, and (4) the preservation of nerve fibers in the sciatic nerve and nerve terminals in the skin.

### Technical considerations of spinal nerve injections: comparison with other approaches

There are two major approaches to introduce gene constructs into the nervous system, as follows: (1) systemic administration and (2) injection into specific areas of the neural axis. The systemic administration of gene constructs is the easiest approach. This is particularly feasible for genes with ubiquitous expression throughout all organs with the ability to cross the blood-nerve barrier^37^. However, the extensive spread of a systemically administered treatment to the brain and peripheral nerves limits its use when a specific target area is required to reduce the adverse effects of ectopic expression. In the experimental treatment of pain, the goal is to express the desired gene constructs in specific regions of the neural axis, e.g., the DRG, to modulate peripheral sensitization^38^. Otherwise, the ectopic expression of pain-modulated opioid receptor in the spinal cord can potentially cause adverse effects in motor function^17^,^39^. Thus, local injection into a specific area is essential under such conditions and is also necessary to study the mechanisms by which a specific region is involved in neuropathic pain.

The specificity of targets is a significant concern as gene expression has been detected in the astrocytes and microglia of the spinal cord following intrathecal injection^4^. In the current report, intrathecal injection with PEI/DNA polyplexes did not induce gene expression in the DRG neurons, which was probably related to a block at the pial meninges. Our study demonstrated that delivery of gene constructs via spinal nerve injection resulted in specific GFP expression in DRG neurons. The absence of GFP expression in the motor neurons and glia of the spinal cord and in the satellite and Schwann cells of the DRG confirmed the specificity of our approach. This is in contrast to a previous report indicating that viral vector transfection methods result in non-specific transfection of glia and neurons^4^,^40^.

Furthermore, the delivery of gene constructs via spinal nerve injection did not damage DRG neurons, as shown by the intact neuronal soma and nerve terminals. Additionally, it did not trigger glial activation in the DRG. The absence of behavioral changes in posture further indicates the preservation of sensory and motor nerve functions.

### Quantifying DRG neurons of different phenotypes

Previous studies have indicated that large-diameter DRG neurons may be preferentially targeted by gene transfer^13^,^16^. This raised the possibility that gene constructs target different subtypes of DRG neurons with different efficiency. To address this issue, we established a method of quantitating the transfection efficiency in subtypes of DRG neurons to understand whether PEI/DNA polyplexes preferentially target a subpopulation of DRG neurons. Previously, immunohistochemical analyses have identified characteristic markers such as neurofilaments, peripherin, neuropeptides of calcitonin gene-related peptide, and substance P^21^,^22^,^27^,^28^,^29^,^30^,^31^. The majority of these studies were used for qualitative analysis, and as such, there remained an issue of how to apply these studies to a quantitative analysis of the entire neuronal population of the DRG. Comparison of DIC microscopy with double immunofluorescent staining of neurofilament and peripherin validated our quantification method as an accurate estimate of the total number of neurons in the DRG. Moreover, this study also demonstrates that the delivery of gene constructs to the DRG via spinal nerve injection labeled all DRG neurons, including those of large and small diameter, without regional preferences.

### Safety of delivering gene constructs to the DRG via spinal nerve injection

Direct intra-DRG injection is a straightforward method to deliver gene constructs into DRG neurons. However, this approach requires extensive surgical procedures to remove significant portions of vertebrae, which could potentially induce inflammation in the DRG and spinal cord^13^. This approach also carries a risk of neuronal injury due to physical trauma and bleeding. Therefore, spinal nerve injection could reduce such risks by avoiding the removal of bony structures, while the surgical risk of intrathecal injection is low. All these concerns regarding the surgical safety and immunogenicity have limited the clinical application of these techniques^13^. In addition, nanoparticles used for non-viral transfection are potentially toxic due to several mechanisms, including membrane destabilization, membrane lysis, and the initiation of inflammatory responses^41^,^42^,^43^,^44^. Based on the analysis of ATF3 expression and glial activation, we demonstrate the safety of non-viral gene constructs in DRG neurons by spinal nerve injection.

In comparison to the disadvantages of nonspecific effects, low transfection efficiency, and risk of nerve injury via injections through intrathecal or intra-DRG routes, spinal nerve injection provides a highly specific, safe, and effective gene therapy route targeting the DRG neurons. In this study, we successfully delivered PEI/DNA polyplexes to DRG neurons. The plasmid sequence, transfection reagents, and delivery route are important factors for achieving successful transfection *in vivo*. The spinal nerve injection procedure does not require the removal of bony structures and thus reduces the risk of nerve injury and inflammation. Furthermore, gene constructs were not diluted with cerebrospinal fluid in the subarachnoid space, which eliminated the concern of widespread off-target transfection. With this novel approach, we aim to facilitate targeted gene therapy intervention in DRG neurons with the long-term goals of enhancing sensory nerve regeneration^45^,^46^ and alleviating neuropathic pain^47^.

## Methods

### Animals

Adult male Sprague-Dawley rats weighing 250–350 g were used in these experiments. Two rats were housed per cage. The rats were fed with a standard laboratory diet and water *ad libitum* and kept at 22 ± 1 °C with a 12-hour light/dark cycle. All procedures were conducted in accordance with the ethical guidelines of the International Association for the Study of Pain (IASP) on the use of laboratory animals in experimental research^48^, and animal protocols have been approved by the Animal Committee of National Taiwan University College of Medicine, Taipei, Taiwan.

### Axotomy by transection of the sciatic nerve

We used axotomy as the positive control for nerve injury by transecting the sciatic nerve^49^. Briefly, rats were anesthetized with isoflurane (induction: 5% and maintenance: 2.5%). The right sciatic nerve was exposed at the mid-thigh level by freeing the adhering fascia between the gluteus and biceps femoris muscles. The sciatic nerve was transected, and 1 mm of the distal stump was removed. The wound was closed with wound clips, and the animals were allowed to recover.

### Polymer conjugate synthesis

Polyethylenimine PEI (*in vivo*-jetPEI™) was purchased from Polyplus-transfection (Polyplus-transfection SA, Illkirch, France). LentiLox 3.7(pLL3.7) is a third-generation lentiviral vector designed for inducing RNA interference in a wide range of cell types, tissues, and organisms^50^,^51^,^52^,^53^. We utilized a LentiLox 3.7 plasmid containing the gene encoding green fluorescent protein (GFP) under the control of a cytomegalovirus (CMV) promoter without extra modification. The relative amounts of vector DNA to carrier were 1.2 μl of transfection reagents per 10 μg of DNA at N/P = 6. The DNA–polymer complexes or PEI alone were diluted with 5% dextrose in water to a total volume of 20 μl and allowed to stand for 15 min at room temperature before use. After incubation, 3 μl of PEI/DNA polyplexes were used for spinal nerve injection and intra-DRG injection, and 10 μl of PEI/DNA polyplexes were used for intrathecal injection.

### Intrathecal injection

Under isoflurane anesthesia (induction: 5% and maintenance: 2.5%), rats were placed in the prone position and a 4 cm incision was made between the L4 and S1 spinal vertebrae. The paraspinal muscles were carefully dissected from their attachments at the L4~S1 levels of the vertebral column. The paraspinal muscles were separated from the spinous processes of the vertebrae. A syringe (Hamilton 80930, Hamilton, Reno, NV) connected to a 26-gauge needle was inserted between the L4~L5 vertebrae at an angle of 60° horizontal to the subarachnoid space of the cauda equina. Access to the intrathecal space was confirmed by reflux of cerebro-spinal fluid (CSF) and the presence of a ‘tail flick’. Ten microliters of PEI/DNA polyplexes containing 0.6 μl transfection reagents and 5 μg DNA were injected intrathecally.

### Lumbar L5 intra-DRG injection

Under isoflurane anesthesia (induction: 5% and maintenance: 2.5%), rats were placed in a prone position. A 5-cm posterior longitudinal skin incision was made at the lumbar segment of the spine. The ipsilateral paraspinal muscles were carefully dissected from their attachments at the L4~S1 levels of the vertebral column. The paraspinal muscles were separated from the spinous processes of the vertebrae. Blunt scissors were used to clean the muscle fascia carefully between the lamina of the L4~L5 vertebrae at the horizontal plane of the iliac crest. After carefully removing the lamina of the ipsilateral L5 vertebrae to expose the L5 spinal nerve and the DRG, PEI/DNA polyplexes (1.5 μg *in vivo*-jetPEI™/3 μl DNA) were injected into the L5 DRG slowly using a syringe (Hamilton 80030, Hamilton, Reno, NV) connected to a 32-gauge needle. After injection, the needle was held at the L5 DRG for 1 min to prevent leakage. Complete hemostasis was confirmed, and the wound was sutured with wound clips.

### Spinal nerve injection of PEI/DNA polyplexes

Under isoflurane anesthesia (induction: 5% and maintenance: 2.5%), rats were placed in a prone position. A 5-cm posterior longitudinal skin incision was made at the lumbar segment of the spine. The ipsilateral paraspinal muscles were carefully dissected from their attachments at the L4~S1 levels of the vertebral column. The paraspinal muscles were separated from the spinous processes of the vertebrae. Blunt scissors were used to clean the muscle fascia carefully between the lamina of the L4~L5 vertebrae at the horizontal plane of the iliac crest and the L5 spinal nerve, which was 2 mm from the L5 DRG. PEI/DNA polyplexes (1.5 μg *in vivo*-jetPEI™/3 μl DNA) were injected into the L5 spinal nerve slowly using a syringe (Hamilton 80030, Hamilton, Reno, NV) connected to a 32-gauge needle. After injection, the needle was held at the L5 spinal nerve for 1 min to prevent leakage. Complete hemostasis was confirmed, and the wound was sutured with wound clips.

### Spinal nerve crush

Under isoflurane anesthesia (induction: 5% and maintenance: 2.5%), rats were placed in a prone position. A 5-cm posterior longitudinal skin incision was made at the lumbar segment of the spine. The ipsilateral paraspinal muscles were carefully dissected from their attachments at the L4~S1 levels of the vertebral column. The paraspinal muscles were separated from the spinous processes of the vertebrae. Blunt scissors were used to clean the muscle fascia carefully between the lamina of the L4~L5 vertebrae at the horizontal plane of the iliac crest and the L5 spinal nerve, which was 2 mm from the L5 DRG. The spinal nerve was crushed with no. 5 forceps (Regine Switzerland, Morbio Inferiore, Switzerland) for 30 seconds. After operation, the animals were cared as those in other groups.

### Animal behavioral tests

Mechanical thresholds and thermal withdrawal latency were measured in behavioral tests.

#### Mechanical threshold

Rats were individually placed in a Plexiglas™ container on metal mesh and allowed to habituate to the new environment for 30 min. A mechanical stimulus was delivered to the plantar surface of the hindpaw from below the floor of the test chamber with a dynamic plantar aesthesiometer (Ugo Basile, Comerio-Varese, Italy). A steel rod (diameter: 0.5 mm) was pushed against the hindpaw with ascending forces of 0 to 50.0 g over a 20-s period. When the animal withdrew its hindpaw, the mechanical stimulus stopped automatically and the force at which the animal withdrew its paw was recorded to the nearest 0.1 g^54^. Each hindpaw was alternatively tested 5 times with a minimal interval of 5 min between measurements. The median of the measurements was used for analysis.

### Thermal withdrawal latency

The Hargreaves-type analgesiometer (Ugo Basile, Comerio-Varese, Italy) was used to measure the hindpaw withdrawal latency upon heat stimulation. Rats were placed in plastic containers on a glass plate. The plantar surface of the hindpaw was directly stimulated with an infrared source below the glass floor. Withdrawal latency was automatically measured from the onset of radiant heat stimulation to the withdrawal of the hindpaw. A maximal latency of 16 s was set to avoid possible tissue damage. Each paw was tested 5 times with a 5-min interval between consecutive trials; the median of 5 withdrawal latencies per side was chosen for analysis.

### Neurophysiological studies

Motor physiology of the sciatic nerve was assessed at one week post-operation. Rats were anesthetized before testing, and the compound muscle action potential (CMAP) was measured using a Nicolet VikingQuest System (Nicolet Biomedical, Madison, WI). Stimulating electrodes were inserted and placed at the sciatic notch to stimulate the sciatic nerve, and recording electrodes were placed on the plantar muscles. The amplitudes of the CMAP on both sides were recorded for analysis.

### Tissue preparation and quantitative multiple-labeling immunofluorescence in DRG neurons

Tissue processing and immunofluorescence were modified from our previous protocols^55^. Animals were terminally anesthetized with isoflurane and perfused with saline followed by fixation with paraformaldehyde/lysine/periodate. Tissues were postfixed for 2 h and subsequently cryoprotected in 30% sucrose in 0.1 M phosphate buffer. Sections (50 μm for spinal cord and 8 μm for the DRG and spinal nerve) were immunostained sequentially and stored at −20 °C. To ensure adequate and systematic sampling, every sixth section was immunostained.

In double- or triple-labeling experiments, the sections were blocked with 0.1% nonfat milk with 0.05% Triton X-100 in 0.5 M Tris buffer (Tris) for 1 h at room temperature and incubated with primary antisera (Table 1) overnight at 4 °C, followed by the appropriate Cy5, Cy3, and fluorescein isothiocyanate (FITC)-conjugated secondary antisera for 1 h (1:100, Jackson ImmunoResearch, West Grove, PA). Sections were photographed using a fluorescence microscope (Axiophot microscope, Carl Zeiss, Heidelberg, Germany). Multiple fluorescence and dark field photographs were processed with AxioVision software (AxioVS40 V 4.8.2.0, Carl Zeiss MicroImage Gmbh, Heidelberg, Germany).

Each DRG section was photographed at 100× magnification with a fluorescence microscope (Axiophot microscope, Carl Zeiss, Heidelberg, Germany) in a systematic fashion to produce a montage of the entire DRG section following established procedures^56^. For neuronal density quantitation, only the area containing neurons was measured. The fluorescence intensity was measured according to a range of 0~255 grayscale units. The faint fluorescence intensity of the naïve DRG neurons was 20~30 grayscale units. Based on these observations, 0~30 grayscale units were designated as background, and 60~255 grayscale units were defined as positive. The positive area of a neuron with a clear round outline was counted as immunoreactive. For Iba1 density quantitation, the intensities of the immunofluorescence signals were subtracted from the background, and each pixel (intensity signal) was equivalent to 0.0625 μm^2^. The area and diameter of the DRG neurons were measured with ImageJ vers. 1.46d software (National Institutes of Health, Bethesda, MD). The density was expressed as neurons/mm^2^ or Iba1 density (μm^2^/mm^2^), and a histogram of the neuronal diameter was plotted.

### Nerve pathology

The processing of nerve pathology followed our established protocol^49^. The sciatic nerves at the gluteal level were collected. Tissues were fixed in 5% glutaraldehyde in 0.1 M PB overnight, postfixed in 2% osmic acid for 2 h at room temperature, dehydrated with a graded series of alcohol, and embedded in Epon 812 resin (Polyscience, Philadelphia, PA). Cross-sections of 1 μm were cut on an ultramicrotome (Leica, Wetzlar, Germany), dried on slides using a hot plate, stained with toluidine blue, and observed under a light microscope.

### Immunohistochemistry in dermal sheets and quantitation of dermal nerve fibers

The plantar skin of the hindpaw was harvested with a 2-mm punch within the L5 spinal nerve dermatome region^57^ from rats without perfusion and incubated in an EDTA solution at 37 °C for 30 min. The dermis was separated from the epidermis and subcutaneous tissues as dermal sheets^58^. Sections of dermal sheets were processed for immunofluorescence labeling as described above with modifications: (1) anti-protein gene product 9.5 (PGP9.5, 1:1000, UltraClone, Isle of Wight, UK), a marker labeling all nerve fibers, was used as the primary antibody and (2) 3,3′-diaminobenzidine (DAB, Sigma, St. Louis, MO) was used as the chromogen.

The quantitation of dermal nerve fibers followed our established protocols^55^. For each dermal sheet, photographs were taken at 200× under a microscope from randomly selected areas covering 30%~35% of the entire dermal sheet section. The intensities of the immunohistochemical signals were subtracted from the background, and each pixel (intensity signal) was equivalent to 0.081 μm^2^. The PGP9.5 nerve fiber area was normalized to the selected area of the dermis and designated as the nerve density (μm^2^/mm^2^).

### Western Blot

Dorsal root ganglia were homogenized in radioimmunoprecipitation assay (RIPA) buffer and sonicated for 40 cycles of 4-s pulses. The protein concentrations were determined using a protein assay kit (BioRad), and samples were stored at 20 °C until further analysis. Protein samples (10 μg of protein/lane) were electrophoresed on a 10% sodium dodecyl sulfate polyacrylamide gel, and the proteins were transferred to PVDF membranes. Membrane strips were blocked for 1 h at room temperature with 5% non-fat milk in Tris-buffered saline (TBS; 150 mM NaCl, 50 mM Tris Base, pH 8.2) containing 0.1% Tween 20 and then incubated overnight at 4 °C with one of the following antibodies, including primary GFP antiserum (goat, 1:1000, R&D Systems, Minneapolis, MN). Immunoblot analyses were performed using horseradish peroxidase-conjugated anti-goat secondary antibodies (1:7500, Promega) and reacted with SuperSignal West Pico Chemiluminescent Substrate (Thermo). The membrane strips were treated with stripping buffer (Thermo) for 30 min and reprobed for beta-actin as an internal control.

### Experimental design and statistical analysis

There were 2 components in this study. In the 1st part of the study, we compared the transfection efficiency and the effects of injection on the DRG among the 3 groups: (1) spinal nerve injection, (2) intra-DRG injection, and (3) intrathecal injection. In the 2nd part of the study, we evaluated the safety of spinal nerve injection using a battery of tests in which spinal nerve injection was compared with sciatic nerve axotomy, which served as the positive control of complete nerve degeneration. There were 2 groups of animals, the spinal nerve injection group (abbreviated as injection group) and the sciatic nerve axotomy group (abbreviated as axotomy group), the latter served as a control. The thermal withdrawal latency and mechanical threshold were evaluated at the following time points: (1) the baseline data before operations as a pretest and (2) at one week post-operation. The animals were randomly assigned to either group. The examiners were blinded to the grouping information when performing the experiments. The grouping information was only decoded when all examinations were complete. There were 5 animals for each laboratory procedure. All data are expressed as the mean ± SEM; a paired Student’s *t* test was performed between two target groups to evaluate significance (p < 0.05).

## Additional Information

**How to cite this article**: Chang, M.-F. *et al*. Effective gene expression in the rat dorsal root ganglia with a non-viral vector delivered via spinal nerve injection. *Sci. Rep.* **6**, 35612; doi: 10.1038/srep35612 (2016).

## Supplementary Material

Supplementary Information

## Figures and Tables

**Table 1:** Table of all antibodies.

Antibody	Purpose	Dilution	Source
Neurofilament (NF)	large neuron	1:500	Covance, Emeryville, CA
peripherin	small neuron	1:500	Chemicon, Temecula, CA
activating transcription factor 3 (ATF3)	nerve injury	1:2000	Santa Cruz Biotechnology, Santa Cruz, CA
Phosphorylated Neurofilament (p-NF)	nerve injury	1:1000	Covance, Emeryville, CA
ionized calcium binding adaptor molecule 1 (Iba1)	macrophage, microglia, satellite cell activation	1:500	Wako, Osaka, Japan
glial fibrillary acidic protein (GFAP)	astrocyte activation	1:500	Chemicon, Temecula, CA
green fluorescent protein (GFP)	transduced gene	1:200	R & D Systems, Minneapolis, MN
