# Supplementary material for: Effective gene expression in the rat dorsal root ganglia with a non-viral vector delivered via spinal nerve injection
Source: Sci Rep. 2016 Oct 17;6:35612. doi: 10.1038/srep35612 (PMC5066268; doi:10.1038/srep35612)
Supplement: Supplementary Information [file srep35612-s1.pdf]

Effective gene expression in the rat dorsal root ganglia with a non-viral vector delivered via spinal nerve injection

Ming-Fong Chang, MS[1], Jung-Hsien Hsieh, MD, PhD[1,2], Hao Chiang, MS[1], Hung-Wei Kan[1], Cho-Min Huang, MS[1], Luke Chellis, BS[9], Bo-Shiou Lin, PhD[4], Shi-Chuen Miaw, PhD[4], Chun-Liang Pan, MD, PhD[5], Chi-Chao Chao, MD, PhD[3], Sung-Tsang Hsieh, MD, PhD[1,6,7,8]

Department of [1]Anatomy and Cell Biology, [4]Graduate Institute of Immunology, [5]Graduate Institute of Molecular Medicine, [6]Graduate Institute of Brain and Mind Science, [8]Graduate Institute of Clinical Medicine, College of Medicine, National Taiwan University, Taipei, 10051, Taiwan

Departments of [2]Surgery and [3]Neurology, [7]Clinical Center for Neuroscience and Behavior, National Taiwan University Hospital, Taipei, Taiwan

Department of [9]Brain and Cognitive Sciences, Massachusetts Institute of Technology, USA

Corresponding authors: Dr. Chi-Chao Chao*, Dr. Chun-Liang Pan* and Dr. Sung-Tsang Hsieh*

Figure S1. Expression pattern of phosphorylated neurofilament and macrophage as injury markers in dorsal root ganglia (DRG) after spinal nerve injection

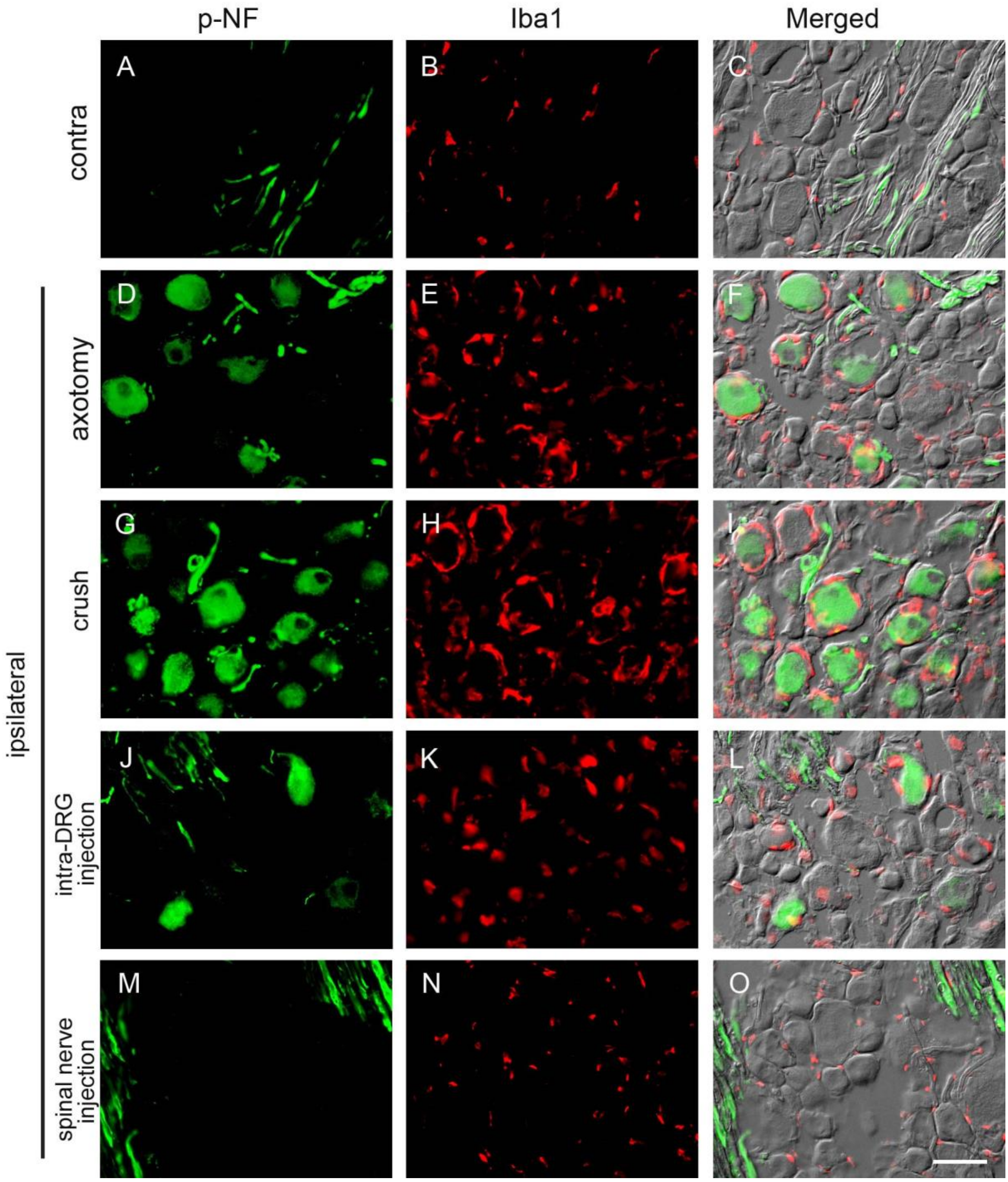

Immunostaining for phosphorylated neurofilament (p-NF) and Iba1 was performed on the dorsal root ganglia one week after operation. Nerve injury induced expression of p-NF in the neuronal cell bodies on the ipsilateral side DRG of the axotomy, crush, and intra-DRG injection groups (Fig. S1D, F, G, I, J, L) compared to the contralateral side and the ipsilateral side of the spinal nerve injection group (Fig. S1A, C, M, O). The Iba1 expression was increased on the ipsilateral DRG of the axotomy, crush, and intra-DRG injection groups (Fig. S1E, F, H, I, K, L) in comparison to the contralateral side and the ipsilateral side of the spinal nerve injection group (Fig. S1B, C, N, O). Bar, 50 µm

Figure S2. Patterns of neurofilament expression and macrophage infiltration as injury markers in the dorsal root ganglia (DRG) after spinal nerve injection

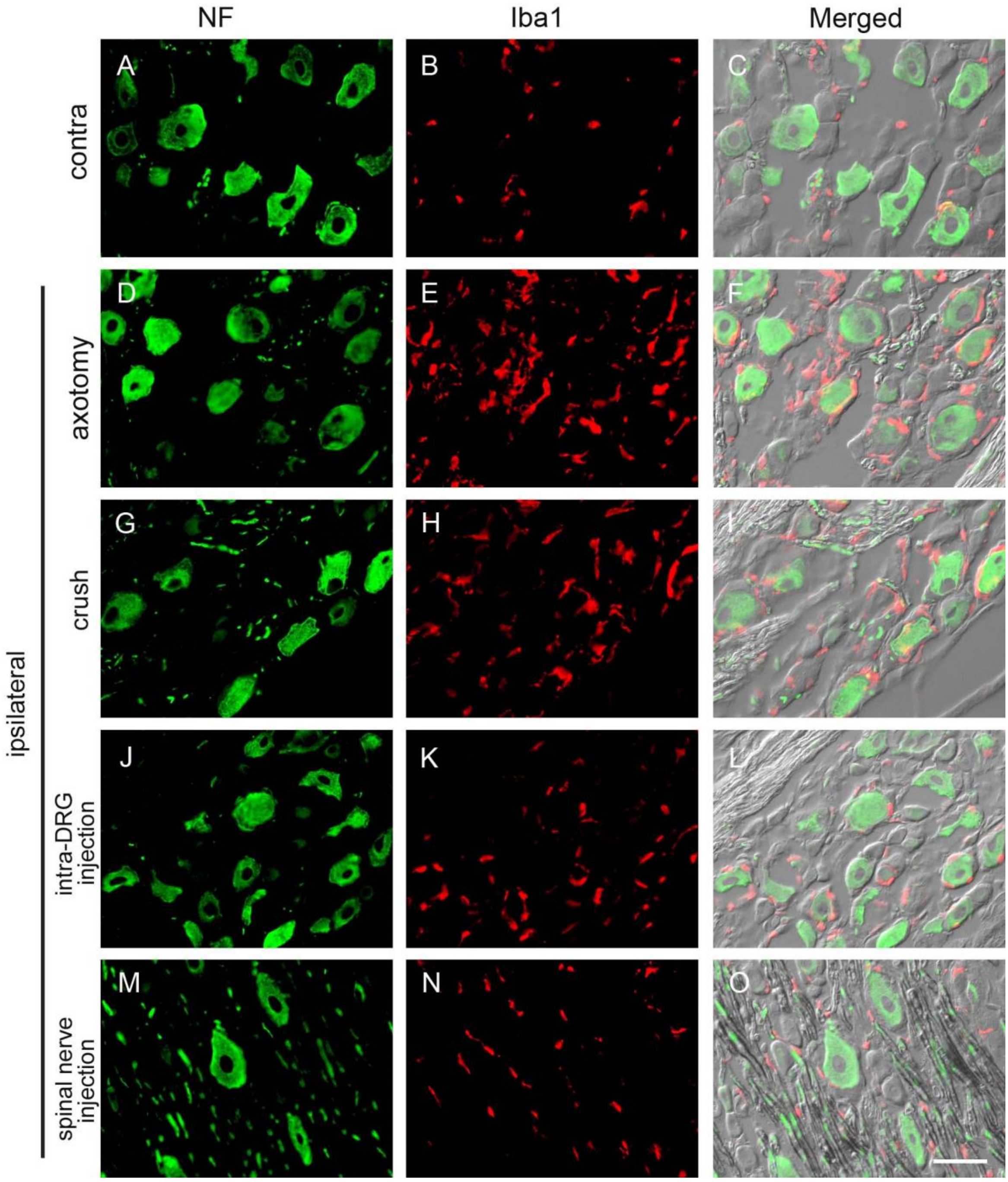

Immunostaining for neurofilament (NF) and Iba1 was performed on the dorsal root ganglia one week after operation. Nerve injury induced eccentric nuclei on the ipsilateral side DRG of the axotomy, crush, and intra-DRG injection groups (Fig. S2D, F, G, I, J, L) compared to the contralateral side and the ipsilateral side of the spinal nerve injection group (Fig. S2A, C, M, O). The Iba1 expression was increased on the ipsilateral DRG of the axotomy, crush, and intra-DRG injection groups (Fig. S2E, F, H, I, K, L) in comparison to the contralateral side and the ipsilateral side of the spinal nerve injection group (Fig. S2B, C, N, O). Bar, 50 μm

Figure S3. GFP expression in the L5 dorsal root ganglia (DRG) was confirmed by immunoblotting after spinal nerve injection.

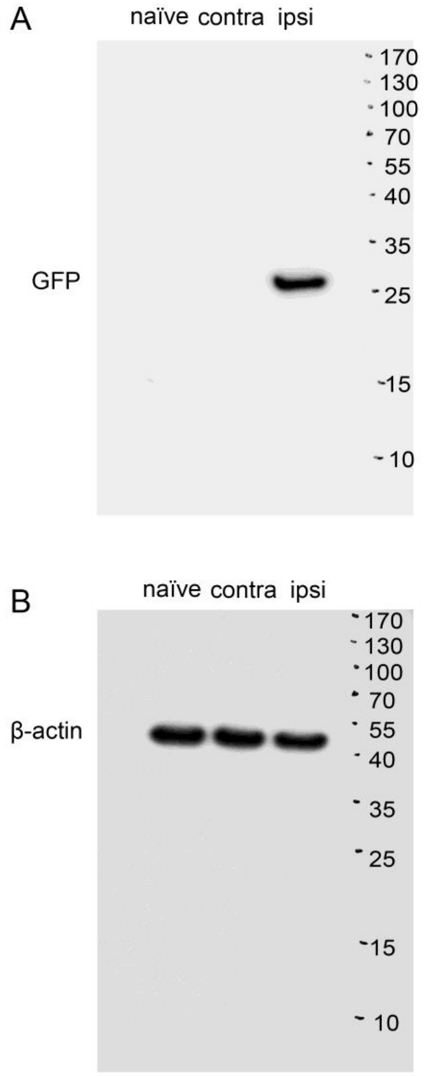

Western blots demonstrated that the GFP expression was specific on the ipsilateral DRG of the spinal nerve injection group compared with the naïve DRG and contralateral side DRG one week after delivering polyethylenimine mixed with DNA plasmids (PEI/DNA polyplexes) that contained the gene encoding green fluorescent protein (Fig. S3A-B).
